# P-1655. Anti-Spike IgG4 and Fc Effector Responses: The Impact of SARS-CoV-2 Vaccine Platform–Specific Priming and Immune Imprinting

**DOI:** 10.1093/ofid/ofaf695.1830

**Published:** 2026-01-11

**Authors:** Raj Kalkeri, Mingzhu Zhu, Shane Cloney-Clark, Anand Parekh, Drew Gorinson, Zhaohui Cai, William Moffitt, Miranda R Cai, Gordon Chau, Tara M Babu, Anna Wald, Louis F Fries, Lisa M Dunkle, Amy W Chung, Joyce S Plested

**Affiliations:** Novavax, Inc., Gaithersburg, Maryland; Novavax, Gaithersburg, Maryland; Novavax, Gaithersburg, Maryland; Novavax, Inc., Gaithersburg, Maryland; Novavax, Inc., Gaithersburg, Maryland; Novavax, Inc., Gaithersburg, Maryland; Novavax, Inc., Gaithersburg, Maryland; Novavax, Inc., Gaithersburg, Maryland; Novavax, Inc., Gaithersburg, Maryland; University of Washington, Seattle, Washington; University of Washington, Seattle, Washington; Novavax, Inc., Gaithersburg, Maryland; Novavax, Inc., Gaithersburg, Maryland; University of Melbourne, at the Peter Doherty Institute for Infection and Immunity, Melbourne, Victoria, Australia; Novavax, Gaithersburg, Maryland

## Abstract

**Background:**

Repeated mRNA SARS-CoV-2 (COVID) vaccination results in large increases in anti-spike (S)-specific IgG4, but this does not occur after repeat vaccination with Matrix-M®-adjuvanted recombinant SARS-CoV-2 S (rS) protein (NVX-CoV2373, Novavax). The clinical relevance of vaccine-induced anti-S IgG4 and its possible reversal remains poorly understood but may be pivotal for this and other future vaccine targets.

**Figure 1:**
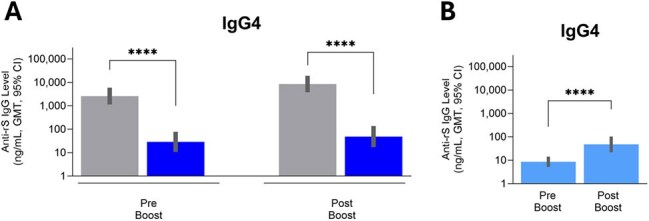
Serum anti-Spike IgG4 concentrations (ng/mL) measured by ELISA. A. Comparison of mRNA vaccine primed 3x (gray) and protein vaccine primed 3x (dark blue) followed by protein vaccine boost subject sera. B. protein vaccine primed 2x subject sera followed by mRNA boost. ****p<0.0001

**Figure:**
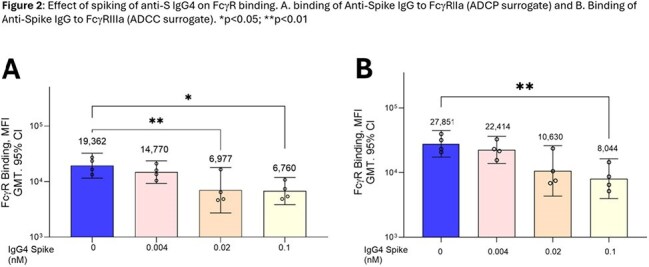

**Methods:**

Sera were randomly selected from study participants who received 1) 3x mRNA vaccine (either BNTb126b2 or mRNA-1273) followed by 1x NVX-CoV2373 (n=19), 2) 4x NVX-CoV2373 (n=18), 3) 2x NVX-CoV2373 followed by 1x BNT162b2 (n=30) and 4) ≥3x mRNA vaccines followed by Matrix-M adjuvanted rS XBB.1.5 (NVX-CoV2601) (n=29). Neutralizing antibodies (nAb), IgG subclass profiles (anti-S IgG1–4), and Fc-mediated effector activities [surrogate ADCP (FcγRIIa binding), ADCC (FcγRIIIa binding), and ADCD (C1q binding)] were assessed. To confirm IgG4 effect, Fc effector activity was measured in sera with low anti-S IgG4 following spiking with polyclonal anti-S IgG4.

**Figure:**
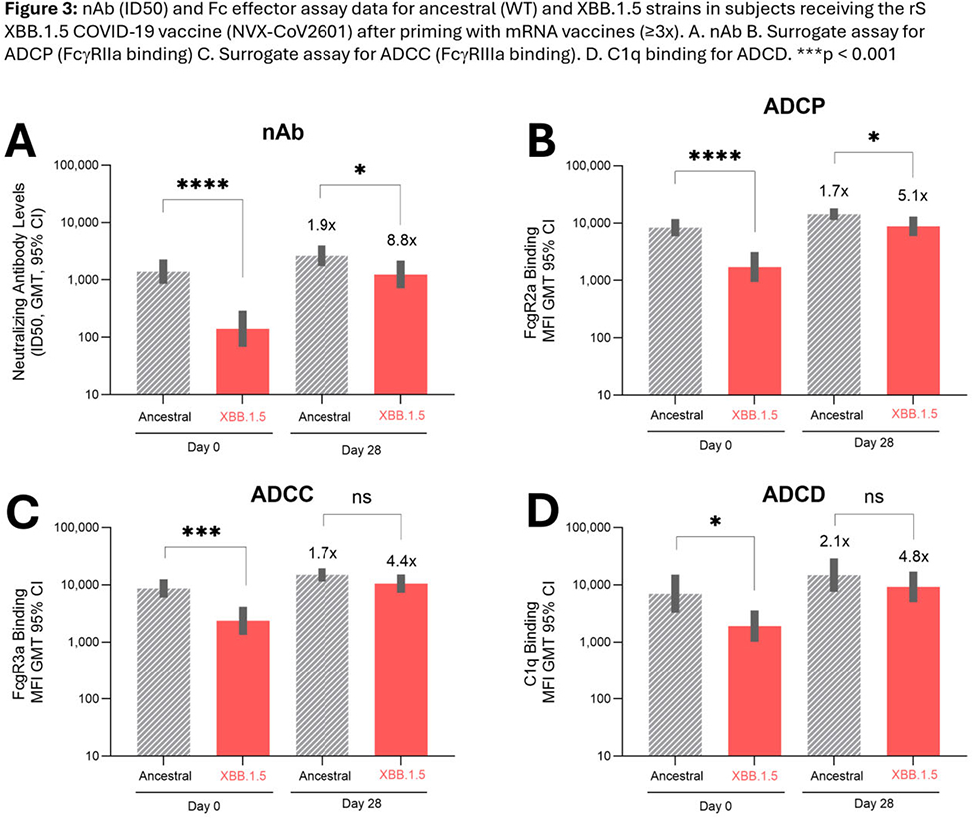

**Results:**

Increases in anti-S IgG4 were only observed in mRNA vaccine recipients and those primed with mRNA vaccines. Anti-S IgG4 was negatively correlated with nAb levels and Fc effector functions. Increasing amounts of added anti-S IgG4 actually lowered Fc effector functions in sera, confirming the negative effect of anti-S IgG4 on Fc effector functions *in vitro*. Boosting with adjuvanted protein-based Novavax XBB.1.5 rS partially overcame anti-S IgG4 and Fc effector function immune imprinting.

**Conclusion:**

These are the first data to demonstrate the importance of immune priming of Fc effector functions with protein-based rS vaccine to reduce the anti-S IgG4 increase observed following mRNA vaccine boosting and to partially overcome mRNA vaccine imprinting with a protein-based rS booster. Study limitations include small sample sizes. Additional work is needed to understand the role of anti-S IgG4-associated reduced nAb levels and reduced Fc effector levels on COVID vaccine effectiveness, immune tolerance to the spike protein and breakthrough infections. These observations can help elucidate differences in immune responses associated with various vaccine platforms.

**Disclosures:**

Raj Kalkeri, PhD, Novavax, Inc.: employee|Novavax, Inc.: Stocks/Bonds (Public Company) Mingzhu Zhu, n/a, Novavax, Inc.: employee|Novavax, Inc.: Stocks/Bonds (Public Company) Shane Cloney-Clark, n/a, Novavax, Inc.: employee|Novavax, Inc.: Stocks/Bonds (Public Company) Anand Parekh, M.Sc., Novavax, Inc.: employee|Novavax, Inc.: Stocks/Bonds (Public Company) Drew Gorinson, B.S., Novavax, Inc.: employee|Novavax, Inc.: Stocks/Bonds (Public Company) Zhaohui Cai, PhD, Novavax, Inc.: employee|Novavax, Inc.: Stocks/Bonds (Public Company) William Moffitt, B.S., Novavax, Inc.: employee|Novavax, Inc.: Stocks/Bonds (Public Company) Miranda R. Cai, PhD, Novavax, Inc.: employee|Novavax, Inc.: Stocks/Bonds (Public Company) Gordon Chau, M.S., Novavax, Inc.: Employee|Novavax, Inc.: Stocks/Bonds (Private Company) Tara M. Babu, MD, MSCI, Sanofi: Advisor/Consultant|Sanofi: Travel support for conference attendance Anna Wald, MD, MPH, Aicuris: Advisor/Consultant|Aicuris: Grant/Research Support|Assembly Sciences: Grant/Research Support|Bayer: Advisor/Consultant|GSK: Advisor/Consultant|GSK: Grant/Research Support|Innovative Molecules: Advisor/Consultant|Merck: Advisor/Consultant|Moderna: Grant/Research Support Louis F. Fries, III, MD, Novavax, Inc.: contractor for Novavax|Novavax, Inc.: Stocks/Bonds (Public Company) Lisa M. Dunkle, MD, Novavax, Inc.: contractor for Novavax|Novavax, Inc.: Stocks/Bonds (Public Company) Amy W. Chung, PhD, Novavax: Grant/Research Support Joyce S. Plested, n/a, Novavax, Inc.: employee|Novavax, Inc.: Stocks/Bonds (Public Company)

